# Feedback-Enhanced Virtual Reality Upper-Limb Training With Body Position Measurement in Healthy Adults: Development and Validation Study

**DOI:** 10.2196/89302

**Published:** 2026-06-16

**Authors:** Naoki Iso, Makoto Suzuki, Takuhiro Okabe, Kazuo Saito, Kilchoon Cho, Takuya Matsumoto, Takako Suzuki, Nanaka Arihara, Junichi Yamamoto

**Affiliations:** 1 Faculty of Health Sciences, Tokyo Kasei University, 2-15-1 Inariyama, Sayama, Saitama, 350-1398, Japan, 81 4-2955-6078; 2 Graduate School of Humanities and Life Sciences, Tokyo Kasei University, 1-18-1, Itabashi, Tokyo, Japan; 3 School of Health and Social Services, Saitama Prefectural University, Koshigaya, Saitama, Japan; 4 Faculty of Health Sciences, Tokyo Kasei University, 2-15-1 Inariyama, Sayama, Saitama, 350-1398, Japan; 5 Faculty of Medical Sciences, Shonan University of Medical Sciences, Yokohama, Kanagawa, Japan; 6 Faculty of Systems Design, Tokyo Metropolitan University, Hino, Tokyo, Japan

**Keywords:** virtual reality, real-time feedback, rehabilitation, motion capture, upper extremity

## Abstract

**Background:**

Virtual reality (VR) systems are increasingly used in rehabilitation to facilitate motor learning by providing visual feedback. However, few studies have validated the motion tracking accuracy of VR devices compared with gold-standard motion capture systems. In particular, validation evidence for upper-limb reaching with commercially available VR tracking setups remains limited.

**Objective:**

This study aimed to evaluate the validity of a custom VR-based rehabilitation system (VRactice) by comparing its motion tracking data with that of a Vicon motion capture system during a goal-directed reaching task in healthy adults.

**Methods:**

This laboratory-based validation study was conducted at Tokyo Kasei University, Sayama Campus, Japan (August-December 2023). Participants were recruited via posted announcements on campus (convenience sampling) and received a 1000 JPY gift card (US $7.00; JPY 142.79=US $1 as of August 1, 2023). A total of 16 healthy participants (n=6, 37.5% male and n=10, 62.5% female participants; mean age 25.3, SD 4.56 years; all right-handed) performed reaching tasks in a VR environment while being tracked simultaneously by both the VRactice system and a Vicon system. Trackers and reflective markers were attached to the hand and elbow to capture 3D coordinates. Each participant performed 10 reaching trials at a frequency of 1 Hz. Data were upsampled to 100 Hz, synchronized, and normalized to the initial position. Valid cycles were identified, and distance time series from the initial position were extracted for the 500-millisecond interval preceding the peak displacement. For each participant, all valid cycles were pooled, and the coefficient of determination (*R*^2^) between VRactice and Vicon trajectories was calculated. Of 160 planned trials (16 participants×10 trials), 4 (2.5%) trials were not recorded; the remaining 156 (97.5%) trials were analyzed without imputation. Statistical significance was evaluated at a 2-sided α level of .05.

**Results:**

Strong agreement between VRactice and Vicon was observed at both the individual and group levels. The *R*^2^ ranged from 0.75 to 0.99 across participants, and all comparisons were statistically significant (*P*<.001). Deviations between the 2 systems remained minimal, confirming that VRactice reliably reproduced the temporal and spatial characteristics of reaching trajectories. At peak displacement, the participant-level mean absolute difference (mean of 10 trials per participant) was 36.5 (SD 29.3) mm (95% CI 20.9‐52.1), suggesting spatial agreement that may be acceptable for upper-limb reaching measurements in this experimental context.

**Conclusions:**

The findings support the validity of VRactice in capturing reaching movements with high spatial accuracy compared with a motion capture system. By providing reliable motion data, VRactice may serve as a useful platform for delivering real-time visual feedback and supporting motor training applications in rehabilitation settings. This study is innovative in that it provides formative validity evidence for a VR-based system that integrates real-time trajectory monitoring with adaptive visual guidance, supporting subsequent clinical implementation and evaluation.

## Introduction

### Background

Motor learning is a fundamental process through which individuals acquire and refine motor skills through practice and experience. This process follows established principles—feedback-based error correction, repetition for consolidation, and task specificity to ensure functional relevance [1-4]. In the domain of rehabilitation, these theories indicate the necessity of precise and structured motor training. According to motor learning theory, individuals adjust motor commands based on the discrepancy between the intended target and the actual movement (eg, the hand trajectory in a reaching task) [5-7]. At the same time, feedforward control—supporting movement planning through sensory predictions or spatial-temporal information before movement execution—has also been recognized as an important factor for efficient motor learning [8,9]. Such anticipatory guidance may allow learners to modify trajectories before large errors occur, leading to more stable and precise performance [6,7,10].

In rehabilitation, reaching is a primary target. Reaching supports many activities of daily living, including eating, dressing, and personal care. Accordingly, for individuals with upper-limb impairments—particularly those recovering from stroke or experiencing age-related decline—difficulty with reaching becomes a major rehabilitation concern [11,12]. In clinical practice, therapists guide and correct movements in real time and adjust the amount and type of instruction according to the patient’s learning stage, enabling effective rehabilitation. Constraint-induced movement therapy [8,13] and task-oriented high-repetition practice [12,14,15] have demonstrated positive outcomes; however, these approaches fundamentally rely on the presence of a therapist and are difficult to implement independently. As an alternative to therapist-delivered guidance, robot-assisted training has become widely used [9,16]. However, most such training primarily provides knowledge of results, while opportunities for continuous knowledge of performance regarding movement quality during execution remain limited [9,17]. Consequently, feedback tends to be intermittent and delayed, making it difficult for patients to detect and correct errors during movement. Moreover, many existing rehabilitation modalities depend largely on feedback control, and mechanisms that progressively adjust feedforward cues according to the learner’s ability or stage of acquisition remain limited in routine practice [17].

Compared with these approaches, virtual reality (VR)–based alternative rehabilitation interventions offer comfort and usability and have become increasingly widespread, yet many implementations emphasize engagement and increased practice dose [16,18-20]. Recent systematic reviews and meta-analyses suggest that adjunctive VR-based rehabilitation can improve upper-limb outcomes after stroke, although intervention content and implementation parameters remain heterogeneous across studies [21]. Furthermore, many rehabilitation and VR-based interventions rely on augmented feedback (knowledge of results and knowledge of performance) delivered during or after movement execution to support error correction [17,22,23]. However, motor learning also depends on predictive feedforward control that shapes the movement plan before execution [6,10,24]. Consistent with the guidance hypothesis, overly frequent or highly guiding augmented feedback can promote dependency and may impair retention when guidance is reduced, highlighting the need for appropriately titrated support [25,26]. Recent validation studies comparing commercially available VR trackers with Vicon have shown that tracking accuracy can vary with setup and task characteristics, underscoring the need for clear validity reporting when proposing VR systems intended for rehabilitation [27]. Although reviews report greater VR-enabled training dose and functional gains, systems that capture and use movement trajectories for both feedback- and feedforward-based learning remain limited [28-31].

Although a small number of studies have examined adaptive cueing in VR settings, the cues used in those systems generally indicate where to move or where to direct attention, rather than providing a visual guide for the movement trajectory itself. In gait rehabilitation as well, adaptive cueing has largely relied on auditory pacing signals. These approaches differ in nature from visual feedforward support. As a result, in the field of upper-limb rehabilitation, systems that adjust visual feedforward cues in real time according to task performance and use them to guide users toward an ideal trajectory remain limited [10,24].

To address this theoretical and technical gap, we developed a VR platform (VRactice) that integrates real-time trajectory monitoring with adaptive guide fading, automatically modulating feedforward cues according to individual motor accuracy. This system complements conventional feedback-based approaches by providing anticipatory feedforward support, thereby promoting more efficient and predictive motor learning [10,24]. The aim of this study was to develop a VR training system (VRactice) that implements real-time adaptive guide fading and to evaluate the agreement of its trajectory measurements with an optical motion capture system under simultaneous recording, providing formative evidence to inform subsequent clinical studies.

### System Development

The VRactice concept and study aims were conceived by the authors (NI and MS). For the exercise task, 13 candidate component movements related to upper-limb activities of daily living were compiled with reference to prior rehabilitation literature [9,15,32] and then selected by consensus among 6 rehabilitation professionals with more than 10 years of clinical experience, who also evaluated the content validity of the candidate component movements. These components included (1) reaching above shoulder level, (2) reaching at shoulder level, and (3) reaching below shoulder level. Based on these evaluations, reaching was identified as the primary training task. The finalized concept and task set were then commissioned to Next System Co, Ltd, which developed the application and implemented real-time motion tracking and system integration.

### System Architecture

**Figure 1.**
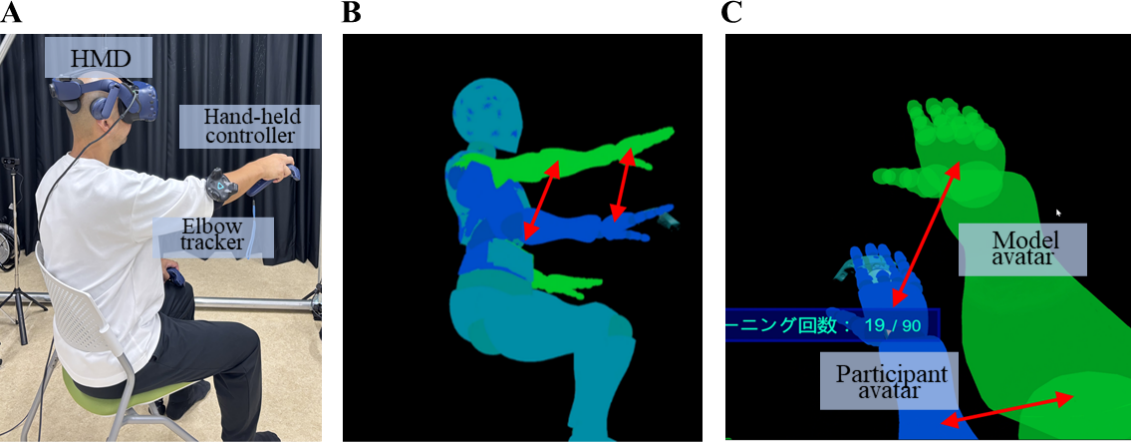
Configuration of the virtual reality system and avatar display during forward-reaching practice in healthy adults (n=16) at Tokyo Kasei University, Sayama Campus (August–December 2023). (A) Experimental setup with the head-mounted display, elbow tracker, and hand-held controller. (B) Operator display showing the model (demonstration) avatar and participant avatar superimposed in the virtual environment. (C) Participant view through the HMD. Arrows indicate the spatial discrepancy (error) between the model and participant avatars.

The system detects 3D body coordinates using VR infrared base stations and trackers and was developed with Unity (Unity Technologies) and the HTC Vive Pro (HTC Corporation; Figure 1A). Two HTC Vive Base Stations 2.0 and an HTC Vive Pro head-mounted display (HMD) were used, together with an elbow tracker and a hand-held controller for upper-limb motion tracking. In VRactice, an ideal model trajectory is displayed and compared with the participant’s ongoing movement to compute spatial error. The system also includes an adaptive guide-fading mechanism that modifies the transparency of the model avatar in real time based on the learner’s performance. Through this combination, users can recognize errors during movement and gradually decrease their reliance on visual cues as their motor skills improve.

Within the VR environment, a green model avatar demonstrates the reference reaching movement, while the participant’s movement is rendered as a blue avatar using data from the HMD, elbow tracker, and hand-held controller (Figure 1). To keep the model and participant avatars anatomically aligned, the participant avatar’s shoulder and elbow joint paths are adjusted to reflect the user’s actual body size and arm length. These adjustments ensure that the visual feedforward cues correspond accurately to the user’s own anatomy, allowing precise trajectory learning during practice.

### Data Recording

The hand position was obtained from the 3D coordinates of the hand-held controller, and the elbow position was obtained from the 3D coordinates of the elbow tracker; both devices were tracked by 2 infrared base stations and streamed to Unity at 70 Hz as time-stamped x, y, z positions in the VR global frame. These coordinates were recorded in the Unity world coordinate system; for the validity analysis, we compared the scalar distance from the initial position over time rather than axis-specific positions. The participant’s coordinates detected in real time were used to compute the distance to the model avatar’s coordinates. These data were recorded in their entirety as time series throughout training.

### Guide Fading (Feedback System)

The participant’s motion data were acquired and displayed as an avatar within the VR environment. The demonstration movement avatar and the participant’s avatar were overlaid for visual comparison. As a preparatory stimulus, the model avatar initiated the movement, prompting the participant to replicate the motion. The discrepancy (distance) between the participant’s and demonstration movements was calculated and averaged per reaching attempt. In this system, “error” (*e*) was defined as the distance between the participant’s hand trajectory and the predefined model trajectory, calculated from the difference between their coordinate positions. The “maximum allowable distance” (*d_max*) represented the therapist-specified tolerance threshold for each trial. In this study, *d_max* was held constant across trials to isolate measurement agreement between VRactice and the Vicon motion capture system; in clinical use, *d_max* can be adjusted by therapists to match the user’s ability and task demands. The feedback evaluation value was determined using the following formula:


$$x\left(\%\right)=100-\left(\frac{e}{d_{max}}\right)\times 100$$

**Figure 2.**
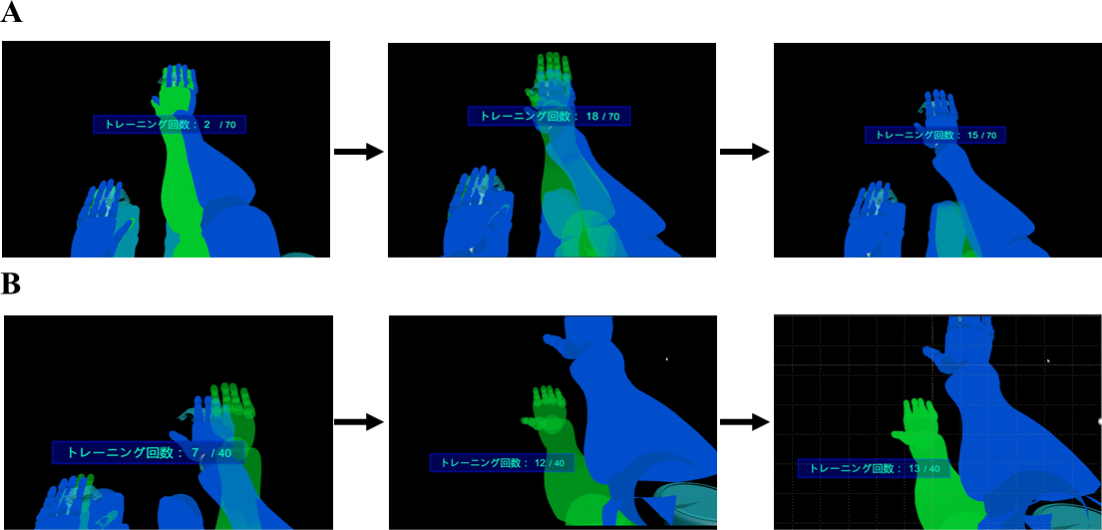
Real-time visual feedback via model avatar transparency during forward-reaching practice. The participant’s avatar was compared with the model avatar to calculate movement errors. Transparency of the model avatar was dynamically adjusted according to the error magnitude, providing real-time visual feedback. (A) When the error was small, the participant’s movement matched the model avatar, which gradually faded. (B) When the error was large, the participant’s movement did not align with the model avatar, which remained opaque and became more prominent to guide the correction.

Increasing the maximum allowable distance resulted in a higher transparency rate, making feedback more forgiving. When the evaluation score exceeded 85%, the model avatar became fully transparent. If the evaluation score remained below 85%, the model avatar did not fade completely; instead, its transparency was maintained so that visual guidance remained available. Transparency adjustments were based on the average error over a predefined number of trials. In addition to the per-trial averaging above, a short moving average across consecutive samples (or trials) was used to stabilize the controller; this smoothed error directly governed the transparency level in real time. When the error decreased (ie, the evaluation score increased), the model avatar became more transparent and visual cues were reduced, whereas when the error remained larger, transparency was held constant to maintain guidance. This behavior is illustrated in Figure 2, where panel A shows increased transparency as the participant’s trajectory approaches the model trajectory, whereas panel B shows preserved opacity when the participant’s hand position deviates from the model avatar’s trajectory.

### Training Customization

The system allows detailed parameter adjustments to tailor training to individual motor abilities, including the reaching direction (9 directions), hand selection (left or right), reaching speed, holding duration at the target, returning speed, intertrial interval, and error-tolerance threshold. The system primarily supports forward-reaching movements; however, users can customize task difficulty by adjusting the direction, speed, number of repetitions, and error-tolerance settings.

## Methods

### Study Design and Setting

This was a laboratory-based validity study conducted at Tokyo Kasei University, Sayama Campus (Neuroscience Practice Room), Japan. Data were collected between August and December 2023. All procedures were completed in a single session per participant.

### Recruitment and Sampling

Participants were recruited through posted announcements on the university campus. We used convenience sampling.

### Ethical Considerations

The study protocol was approved by the Tokyo Kasei University Ethics Committee (SKE2021-25) and conducted in accordance with the Declaration of Helsinki. All participants provided written informed consent prior to participation. Data were deidentified prior to analysis and stored on access-restricted devices. Participants received a 1000 JPY gift card as compensation for their time. No images in the manuscript or supplementary materials permit identification of individual participants; if any identifiable images are included, explicit permission for publication was obtained.

### Safety Monitoring

Because participants repeatedly performed reaching movements while wearing a HMD and trackers, we monitored discomfort and fatigue throughout the session. Before starting the task, participants were asked to move their hands and head while wearing the devices to confirm the absence of nausea or other discomfort. During data collection, we monitored the marker attachment sites for itching and checked for signs of discomfort related to wearing the VR equipment. If any adverse symptoms were observed, the devices were removed immediately and the session was stopped. After completion, reflective markers and VR devices were removed promptly. Participants were informed that they could pause or stop the experiment at any time without penalty, and subjective fatigue was checked during the session. If a participant developed significant symptoms, the session was terminated and the participant was instructed to rest; if needed, evaluation by the campus health office or a nearby medical facility was arranged.

### Participants

The participants were 6 right-handed adult male participants and 10 female participants. Participants were recruited between August and December 2023. The mean age was 25.3 (SD 4.56) years. According to the Edinburgh Handedness Inventory [33], all participants were right-hand dominant (laterality quotient: mean 91.29, SD 7.88). All participants reported no history of neurological or musculoskeletal disorders affecting the upper limb and had normal or corrected-to-normal vision.

### Experimental Setting

**Figure 3.**
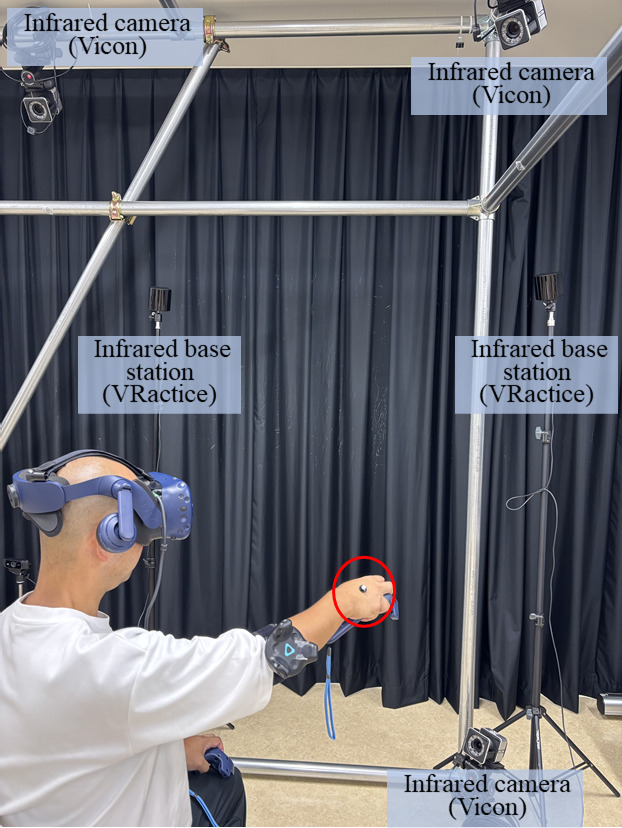
Simultaneous measurement setup for VRactice and the motion capture reference system in healthy adults (n=16). For VRactice, trackers were attached to the participant’s hand and elbow, and 2 infrared base stations recorded 3D position coordinates. For the motion capture reference (Vicon Motion Systems), reflective markers (indicated by red circles) were placed on the participant’s hand and back, and 3 infrared cameras recorded their spatial positions.

For the VRactice task, a tracker was attached at the elbow and the hand-held controller was used to capture hand position, and 2 infrared base stations were used to measure the position coordinates of the trackers to project an avatar in VR. For the motion capture device, Vicon (Vicon Motion Systems) was used. Reflective markers were attached to the center of the dorsum of the hand and upper back, and the position coordinates were measured with 3 infrared cameras (Figure 3).

### Experimental Motor Task

The motor task in the experiment was a reaching task wherein the participant extended their hands forward in accordance with a model movement in VR; the task was repeated for 10 cycles at a frequency of 1 Hz with a 1-second break in between. In this study, the transparency of the exemplar motion was kept constant regardless of the error to verify the consistency of the body coordinates between the VRactice and the motion capture device.

### Data Processing and Analysis

The VRactice data were first interpolated and upsampled to 100 Hz to match the temporal resolution of the Vicon system. Of 160 planned trials (16 participants×10 trials), 4 (2.5%) trials were not recorded; the remaining 156 (97.5%) trials were analyzed without imputation. For each trial, the distance from the initial position was calculated for both VRactice and Vicon coordinates. To identify valid reach cycles, we defined a cycle as valid when displacement from the initial position exceeded 100 mm. Within each valid cycle, the peak displacement was identified, and the 500-millisecond segment preceding the peak was extracted for agreement evaluation. Time-series metrics were computed using all available cycles (P04: 7; P07: 9; others: 10).

For each participant, all valid cycles (available repetitions) were pooled, and the coefficient of determination (*R*^2^) between VRactice and Vicon was computed on the pooled series to evaluate agreement. For each participant, and for each of the 10 reach trials, the distance from the initial position to peak displacement was computed separately for VRactice and Vicon. For each trial, the absolute difference between these 2 distances was then calculated. The 10 per-trial differences were then averaged to obtain a participant-level mean, and these participant-level means were subsequently averaged across participants to yield the overall mean difference between the 2 systems. We did not apply post hoc outlier exclusion; instead, variability in the observed differences was summarized using SD and 95% CIs.

## Results

**Figure 4.**
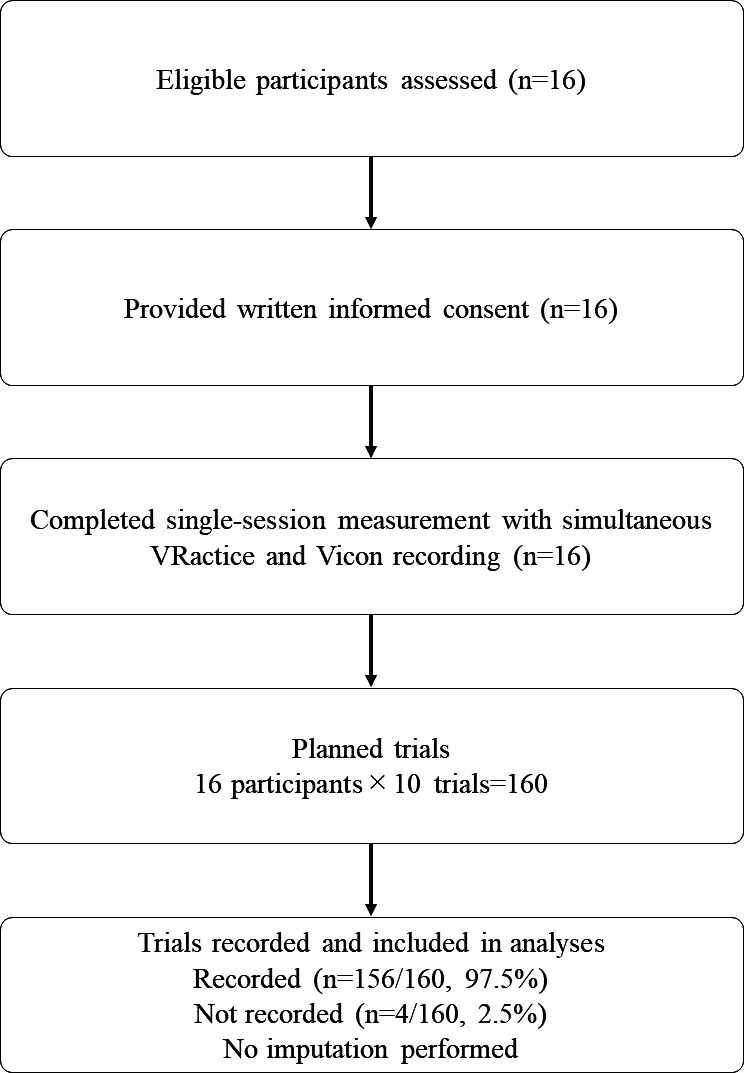
Participant flow diagram.

**Figure 5.**
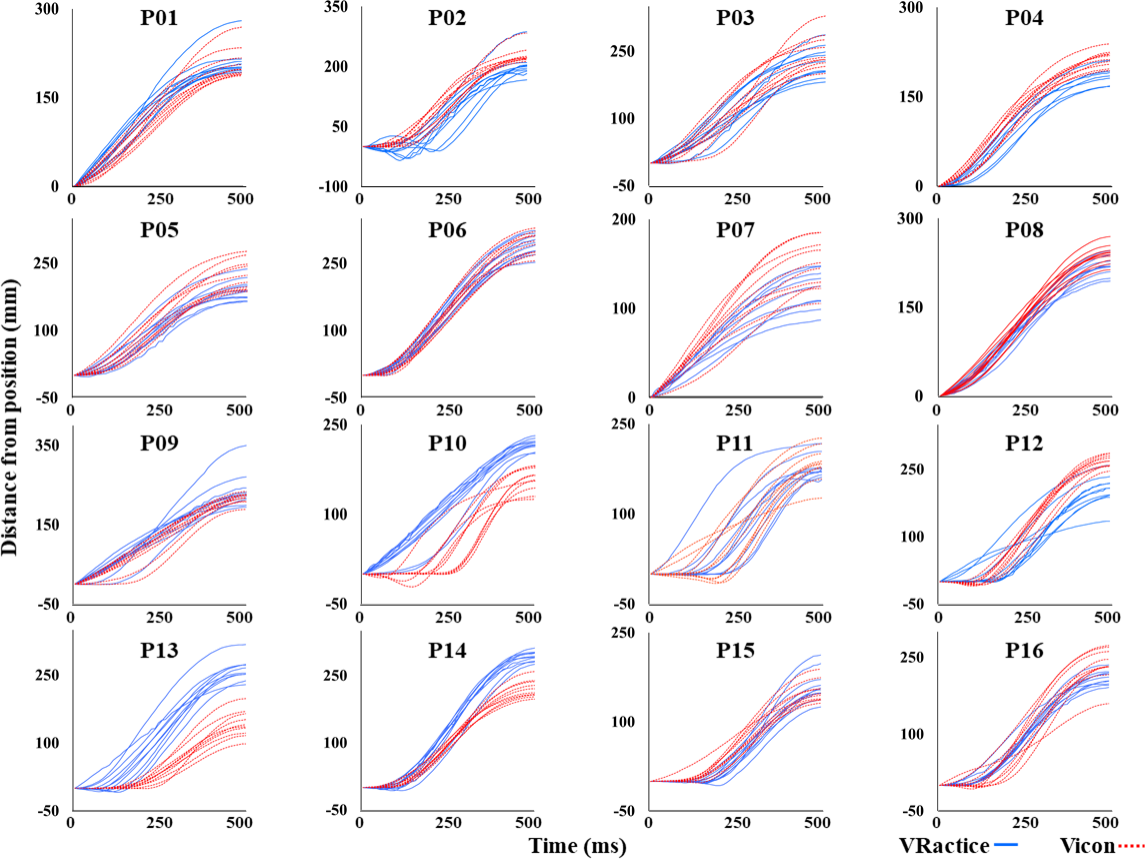
Individual distance time-series trajectories from VRactice and Vicon (P01-P16). Both datasets were baseline-corrected to 0 at the initial value. Blue solid lines indicate VRactice data and red dashed lines indicate Vicon data. Each panel represents 1 participant (500-ms prepeak segment used for agreement evaluation).

**Figure 6.**
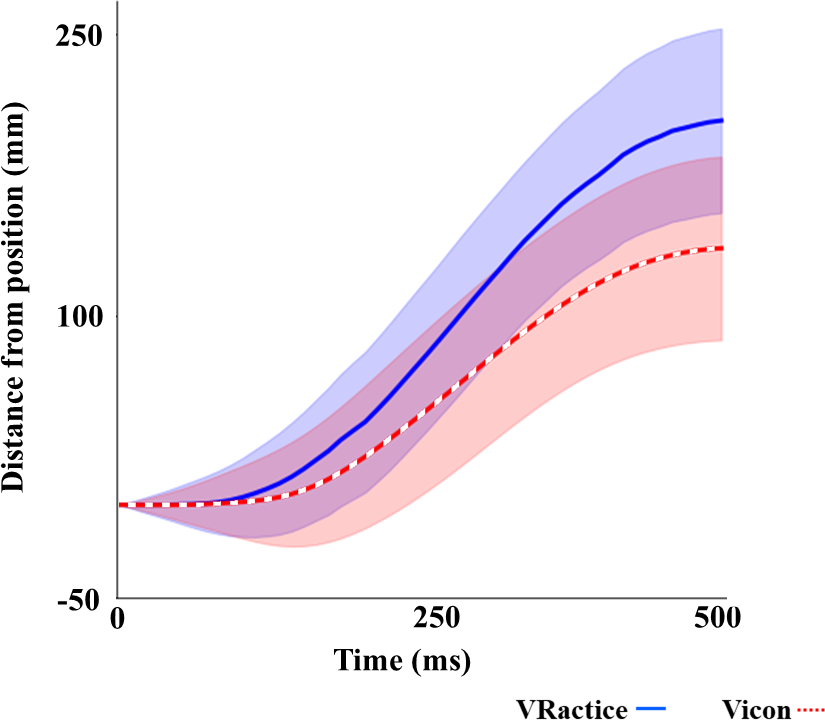
Group-mean distance trajectories from VRactice and Vicon (n=16). VRactice and Vicon are shown as blue solid and red dashed lines, respectively; shaded areas indicate +1 to –1 SD for the 500-millisecond prepeak segment.

Participant flow is shown in Figure 4. Across 16 participants (10 trials each), agreement between VRactice and Vicon was high when evaluated on the 500-millisecond prepeak segment of the distance-from-initial-position trajectories (Figure 5). Figure 6 shows the corresponding group-mean profiles (mean, SD).

Participant-level *R*^2^ ranged from 0.75 to 0.99, and all correlations were statistically significant (*P*<.001; Table 1). These results indicate that VRactice reliably reproduced the temporal and spatial patterns captured by Vicon. At the point of peak displacement, the participant-level mean absolute difference (mean of available trials per participant) was 36.5 (SD 29.3) mm (95% CI 20.9‐52.1; Table 2).

**Table 1.** Participant-level coefficients of determination between VRactice and Vicon^a^.

Participants	*R* ^2^	*P* value
P01	0.981	<.001
P02	0.809	<.001
P03	0.995	<.001
P04	0.901	<.001
P05	0.982	<.001
P06	0.995	<.001
P07	0.762	<.001
P08	0.998	<.001
P09	0.797	<.001
P10	0.753	<.001
P11	0.849	<.001
P12	0.837	<.001
P13	0.883	<.001
P14	0.972	<.001
P15	0.920	<.001
P16	0.911	<.001

a The coefficients of determination (*R*2) and *P* values between VRactice and Vicon. For each participant, all valid cycles were pooled to compute the *R*2.

**Table 2.** Mean absolute displacement differences between VRactice and Vicon at peak displacement^a^.

Participant	Difference (mm), mean (SD)
P01	10.4 (11.9)
P02	38.2 (40.1)
P03	23.6 (8.8)
P04	19.2 (2.2)
P05	32.3 (12.2)
P06	3.4 (7.8)
P07	26.6 (2.6)
P08	20.1 (2.9)
P09	30.4 (53.3)
P10	58.9 (21.7)
P11	17.8 (21.3)
P12	62.9 (38.1)
P13	119.5 (26.2)
P14	71.6 (18.7)
P15	14.2 (15.4)
P16	34.3 (30.8)
Average	36.5 (29.3)

a Values are presented as participant-level means (mean of 10 trials per participant). Across participants (n=16), the total mean was 36.5 (SD 29.3); 95% CI 20.9-52.1.

## Discussion

### Principal Findings

This laboratory-based validity study evaluated the agreement between VRactice and an optical motion capture reference (Vicon) during a goal-directed reaching task in healthy adults.

This study developed an adaptive VR system centered on feedforward visual guidance that proactively assists trajectory correction before errors emerge, while also integrating real-time error-based feedback as a complementary mechanism. VRactice is characterized by presenting within the VR environment a model avatar that serves as a model for accurate movement, allowing participants to repeatedly practice reaching while aligning their actions with the model avatar. Furthermore, VRactice provides not only real-time, error-based feedback but also a feedforward-based cue presentation by fading the display of the model avatar based on the distance (ie, error) between the model avatar’s movement and the participant’s movement, thereby enabling anticipatory trajectory correction before the error becomes overt.

In the present dataset, agreement between VRactice and Vicon was high on the prespecified 500-millisecond prepeak segment, and the participant-level mean absolute difference at peak displacement was 36.5 (SD 29.3) mm (95% CI 20.9‐52.1).

Taken together, these results suggest that VRactice can capture reaching-related spatial trajectories in close agreement with optical motion capture under simultaneous recording.

### Comparison With Existing Rehabilitation Technologies

By comparison, training approaches that integrate robotics with surface electromyography, functional electrical stimulation, or electroencephalography-based brain-computer interfaces can estimate muscular or cortical states in real time and provide temporally precise feedback. Randomized trials and meta-analyses have reported promising poststroke motor improvements with such approaches [16,34-37]. However, despite their technical sophistication, broad clinical adoption is often limited by device complexity, maintenance burden, and challenges related to comfort and usability [38,39].

Given these limitations, VR-based systems—and particularly VRactice—may provide a more practical and accessible solution [40]. From this standpoint, VRactice extends these advantages further by integrating both feedforward cueing and error-based feedback within a single, low-burden platform.

### Interpretation in the Context of Motor Learning Theory

Although many conventional VR rehabilitation systems use off-the-shelf trackers to deliver task-based training and provide performance feedback after movement execution, VRactice uses the same pragmatic hardware foundation yet differs in important ways [5-7]. The system is designed to detect movement accurately and provide real-time feedback. Instead of relying on post hoc knowledge of results, it delivers continuous visual guidance (knowledge of performance) with transparency adaptively faded to the participant’s ability, enabling guided practice. Importantly, VRactice not only implements error-based feedback but also places its core emphasis on a feedforward-based cue presentation that proactively assists users before errors become evident. By automatically modulating visual guidance depending on performance, the system supports anticipatory trajectory control and thus enhances the efficiency of motor learning.

Implemented as adaptive fading that tapers assistance as proficiency increases, this approach aligns with the guidance hypothesis framework of optimizing support [25,26]. Beyond its adaptive feedback design, VRactice also incorporates features for individualized therapy. To facilitate such individualized adaptation, VRactice allows explicit setting of parameters such as reaching direction, speed, hold, return timing, and the maximum allowable distance threshold, enabling therapists to fine-tune task difficulty according to the participant’s proficiency and fatigue. For example, setting a strict error tolerance weights training toward fine distal control of the hand, whereas a more lenient setting weights training toward acquisition of gross reaching strategies. These configurations preserve the cost and ease of installation and deployment advantages of commercial VR hardware while enabling high-quality reaching practice in clinical settings, aligning with implementation benefits noted in prior literature [28,29,35].

VR-based rehabilitation has been shown to improve motivation and adherence compared with conventional approaches [41]. The adaptive guide-fading behavior in VRactice aligns with principles of minimizing compensatory movement and promoting restoration of appropriate motor patterns, particularly relevant in poststroke rehabilitation [32]. Furthermore, because feedforward control declines with aging and neuromuscular disorders, VRactice’s real-time modulation of proactive cues may provide clinical value for diverse patient populations [10-12].

### Methodological Considerations

To further validate the accuracy of VRactice’s guidance, we compared its measurements with those of an existing 3D motion analysis system. Moreover, the observed mean difference between the existing 3D motion analysis system and VRactice at the point of peak displacement supports the view that the trajectory guidance and measurements provided are both accurate and clinically useful in this experimental setup. In other words, this level of accuracy ensures that both feedforward guidance and error-based correction are grounded in reliable spatial information. While few-millimeter verification may be necessary for fingertip precision tasks, in clinical contexts focused on facilitating learning of upper-limb reaching, it is important to guide the limb along an appropriate trajectory toward the target [30,34]. Accordingly, the several-centimeter error observed in this study is within the range reported in prior validation studies of existing VR systems [42-44].

Because VR-based tracking and optical motion capture differ in sampling characteristics and synchronization, this study prioritized spatial agreement using a phase-aligned 500-millisecond prepeak segment rather than attempting full-cycle temporal alignment from movement onset. Therefore, we did not perform statistical testing of time-course differences over the entire reach-return cycle. Future work should refine synchronization and evaluate agreement over the full movement cycle, including onset alignment and time-course differences.

These findings support the validity of VRactice’s design philosophy, which emphasizes not only post hoc feedback correction but also proactive feedforward cueing tailored to individual ability for shaping accurate movement trajectories [10,17,24,45].

### Limitations

Several limitations should be noted. First, the sample size was modest and limited to healthy adults, which restricts generalizability to patient populations and real-world clinical settings. Second, we evaluated agreement using a peak-aligned prepeak segment rather than the full reach-return cycle and therefore did not quantify differences across the entire movement time course from onset to return. We also did not conduct hypothesis testing of time-course differences over the entire movement cycle. Third, the reported validity evidence is specific to the present hardware configuration and experimental setup, and tracking accuracy may vary depending on task characteristics and setup [42-44]. Finally, this validation study did not evaluate clinical effectiveness or learning outcomes; subsequent studies should assess whether adaptive guide fading improves retention and functional performance in clinical populations.

In addition to VR, augmented reality has also been explored as a modality for delivering perceptual cues and promoting safer movement behaviors; therefore, future work should compare whether similar cue-based strategies can be implemented and validated across VR and augmented reality systems [46].

### Conclusions

VRactice is an adaptive VR reaching-training system centered on feedforward cue presentation, while maintaining clinically reliable spatial accuracy in tracking movement. By integrating real-time, performance-contingent modulation of visual guidance, the system may enhance motor learning and contribute to improved rehabilitation outcomes. Future work should optimize clinical implementation and extend applicability to broader patient groups.

In summary, these findings provide formative validity evidence supporting the feasibility of VRactice for trajectory-based motion monitoring and guided reaching practice in rehabilitation-oriented settings.

## Abbreviations

HMD: head-mounted display
VR: virtual reality
